# A Case of Takotsubo Cardiomyopathy in a Patient With Coronary Artery Fistula

**DOI:** 10.7759/cureus.26896

**Published:** 2022-07-15

**Authors:** Ngoda Manongi, Sijun Kim

**Affiliations:** 1 Internal Medicine, NewYork-Presbyterian Queens, Flushing, USA; 2 Cardiology, NewYork-Presbyterian Queens, Flushing, USA

**Keywords:** transthoracic echocardiography, acute coronary syndrome, non-obstructive coronary artery disease, coronary artery fistula (caf), takotsubo cardiomyopathy (ttc)

## Abstract

Takotsubo cardiomyopathy (TTC) is also known as stress-induced cardiomyopathy and mimics acute coronary syndrome in the setting of non-obstructive coronary artery disease. It is associated with reversible left ventricular apical, mid, and/or basal wall motion abnormalities. A coronary artery fistula (CAF) is a connection between one or more of the coronary arteries and the cardiac chamber or great vessel. We present a case of an elderly woman who presented with chest pain and was found to have non-obstructive coronary artery disease with wall motion abnormality pattern consistent with TTC and multiple CAF involving the left circumflex coronary artery and pulmonary artery. This case highlights a rare association between two uncommon entities.

## Introduction

Takotsubo cardiomyopathy (TTC), also known as stress-induced cardiomyopathy, is a reversible cardiomyopathy characterized by left ventricular dysfunction and ballooning of the left ventricular apex in the absence of obstructive coronary artery disease. TTC typically occurs in postmenopausal women in the setting of acute emotional or physical stress and clinically presents similar to acute coronary syndrome [1]. The pathophysiology of TTC is not well established but is thought to involve coronary vasospasm, microvascular dysfunction, and catecholamine toxicity [2]. Coronary artery fistula (CAF) is characterized by anomalous communication between the coronary artery and the heart chamber or the great vessels. CAF is thought to occur in approximately 0.1% to 0.2% of all patients who underwent invasive coronary angiography [3]. The majority of CAF start from the right coronary artery or left anterior descending artery followed by the left circumflex, diagonal, and left main coronary artery. The clinical significance of CAF is determined by the site of origin, termination, and magnitude of shunting.

## Case presentation

**Figure 1:**
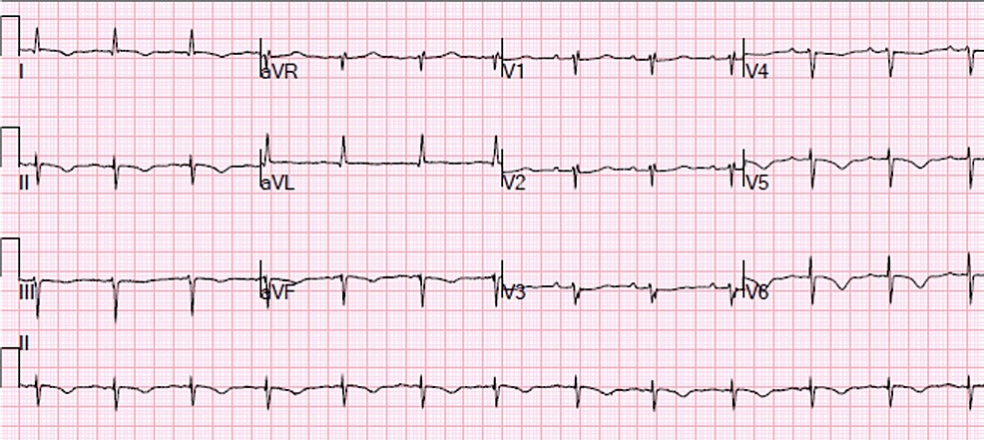
Twelve-lead ECG showing possible ST-segment elevation in V5-V6, poor R wave progression, and T wave inversion in I, II, III, aVF, and V4-V6.

**Figure 2:**
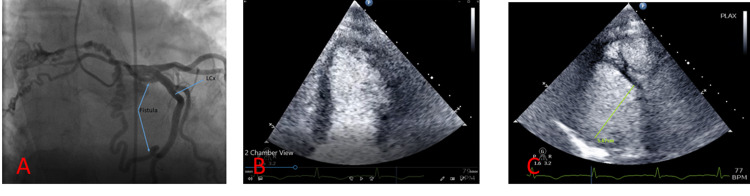
(A) Coronary angiography showing fistulous connections between the left circumflex (LCx) and the pulmonary artery in the absence of coronary artery disease. (B) Contrast echocardiography showing regional wall motion abnormality with severe hypokinesis of the apex and mid left ventricular segments with preservation of the basal segments. (C) Contrast echocardiogram demonstrating moderately dilated left ventricle (left ventricular internal diameter in diastole = 5.8 cm).

**Figure 3:**
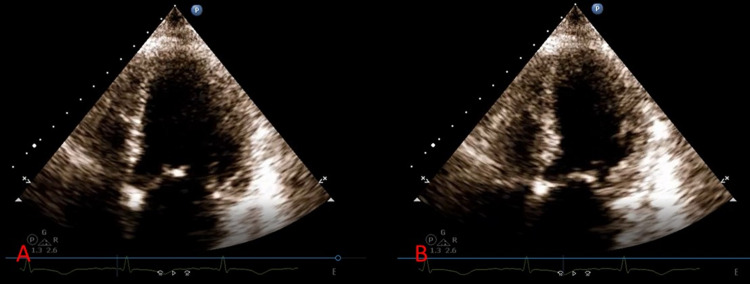
Apical four-chamber view. (A) Left ventricular end-diastole and (B) left ventricular end-systole. Severe hypokinesis of the apex and mid left ventricular segments with preservation of the basal segments.

A 76-year-old female with a past medical history of paroxysmal atrial fibrillation status post ablation presented to the emergency room with complaints of chest pain and shortness of breath. The pain was radiating to the left arm, 7/10 in intensity, substernal, pressure-like, and made worse with exertion. The patient appeared pale, diaphoretic, and cool to touch. Of note, the patient stated that for the last three months, she had been experiencing a lot of anxiety and stress from work and at home. The patient denied toxic habits including illicit drug use such as cocaine. In the ED, she was afebrile, with a heart rate of 89 beats per minute, blood pressure of 154/103 mmHg, respiratory rate of 18 breaths per minute, and oxygen saturation (SPO2) of 96% on a 4 L nasal cannula. Blood work was significant for WBC of 15.63 K/uL, positive urinalysis, and troponin T of 0.322 ng/mL (Table 1). ECG showed possible ST elevations in V5-V6, T wave inversion in leads I, II, III, aVF, and V4-V6, and poor R wave progression (Figure 1). Due to concerns for ST-elevation myocardial infarction, the patient was immediately taken to the cath lab. A left heart catheterization revealed no significant coronary artery disease but revealed two fistulous connections between the left circumflex artery and pulmonary artery (Figure 2A). Also, contrast echocardiography showed regional wall motion abnormality with severe hypokinesis of the apex and mid left ventricular segments with preservation of the basal segments and moderately dilated left ventricle (Figures 2B, 2C). Transthoracic echocardiogram showed moderately dilated left ventricle (left ventricular internal diameter in diastole = 5.8 cm) with mild systolic dysfunction (left ventricle ejection fraction = 40-45%), pulmonary hypertension (pulmonary artery systolic pressure = 50-55 mmHg), and severe hypokinesis of the apex and mid left ventricular segments with hypermobile basal segments, consistent with TTC (Figures 3A, 3B).

**Table 1:** Laboratory parameters for the duration of the patient's hospital stay.

	Admission	8 hours after admission	Hospital day 2	Hospital day 3	Reference range
WBC (in K/uL)	15.63		11.36	10.49	4.80-10.80
Troponin (in ng/mL)	0.320	0.289	0.255		0.010-0.030
Pro-brain natriuretic peptide (in pg/mL)	4010				0-450
Urinalysis					
Color	Orange			Yellow	
Appearance	Turbid			Clear	
WBC	>100			10	
Bacteria	Positive			Negative	
Nitrite	Positive			Negative	
Leukocyte esterase	Moderate			Negative	
Squamous epithelial cells	3			3	

## Discussion

Coronary-to-pulmonary artery fistula (CPAF) is the most common type of CAF seen on coronary computed tomography angiography (CCTA) [4]. The clinical significance of a CAF depends on the resistance of the fistulous connection and the difference in pressures between the coronary artery and the site where the fistula drains. Most CPAFs have negligible shunting of blood due to their small size. However, some can be large and lead to a sizable shunting of blood from the coronary circulation to the low-pressure vascular bed, resulting in pulmonary hypertension and left volume overload. This can be detected indirectly by echocardiography with signs of left chamber enlargement. In rare cases, significant diastolic run-off can divert blood away from the coronary and myocardial microcirculation and lead to symptoms of ischemia (coronary-steal phenomenon). Accurate imaging with CCTA or magnetic resonance imaging (MRI) is helpful to assess the receiving chamber or vessel and to confirm the entry site as well as the patency of the shunt.

There are no established guidelines for the management of patients with CAF. While small asymptomatic fistula can be safely monitored, large fistulae invariably lead to complications. Many experts argue for invasive management if the pulmonary to systemic flow ratio exceeds 1.5:1, or in cases of aneurysmal degeneration [5]. CAF can be closed surgically (ligation and/or fistula excised) or percutaneously (fibered coils, vascular plug, balloons, and alcohol injection) and is dependent on its morphology, its course, tortuosity, and the presence of aneurysmal dilation [6].

Our patient was initially managed with acute coronary syndrome (ACS) protocol with aspirin 325 mg, clopidogrel 600 mg, metoprolol tartrate 12.5 mg, and atorvastatin 80 mg. For the urinary tract infection (UTI), the patient received ceftriaxone 1 g daily for five days with improvement in WBC immediately (Table 1). Other etiologies such as pheochromocytoma and myocarditis were considered. After carefully evaluating the patient's outpatient medical records, we learned that patient's blood pressure (BP) has always been normal and actually on the lower end of normal. It appears that her elevated BP during admission was likely due to stress. We decided not to test for pheochromocytoma in the patient. In addition, given the improvements in WBC after treatment for UTI, we decided that myocarditis was unlikely. Following cardiac catheterization, the patient remained free of symptoms and did not require further intervention. Although our patient had signs of left ventricular volume enlargement and pulmonary hypertension by echocardiography, invasive hemodynamic and advanced cardiac imaging was not performed to assess the significance of the CPAF. The patient was diagnosed with TTC and a coincidental CPAF. As such, therapy was directed toward the treatment of mild systolic dysfunction, and serial monitoring of the fistula with CT imaging and echocardiogram to assess for progressive chamber enlargement or reduction in ejection fraction was recommended in the outpatient setting. The patient was discharged home on hospital day four and remains symptom-free at one- and six-month follow-up.

## Conclusions

CAFs are rare cardiac anomalies, but they should always be part of the differential diagnosis of symptoms of chest pain and dyspnea, especially in patients without significant risk factors for acquired cardiac disease. They can give rise to a variety of symptoms because of their hemodynamic consequences or complications. Our case illuminates the important association between two rare conditions and highlights that CAF can lead to left-to-right over-circulation and result in right-sided remodeling and pulmonary hypertension.
